# Risk Factors for Middle Ear Barotrauma in Patients with Carbon Monoxide Poisoning Undergoing Monoplace Hyperbaric Oxygen Therapy: A Retrospective Cohort Study

**DOI:** 10.3390/jcm14092984

**Published:** 2025-04-25

**Authors:** Jung-Ho Lee, Hyoung-Youn Lee, Kyung-Hoon Sun, Tag Heo, Sung-Min Lee

**Affiliations:** 1 Department of Emergency Medicine, Chonnam National University Hospital, 42 Jebong-ro, Dong-gu, Gwangju 61469, Republic of Korea; sw97220501@naver.com (J.-H.L.); docheo@hanmail.net (T.H.); 2 Trauma Center, Chonnam National University Hospital, 42 Jebong-ro, Dong-gu, Gwangju 61469, Republic of Korea; apostle09@naver.com; 3 Trauma Center, Chonnam National University Medical School, 42 Jebong-ro, Dong-gu, Gwangju 61469, Republic of Korea; 4 Department of Emergency Medicine, College of Medicine, Chosun University, 365 Pilmun-daero, Dong-gu, Gwangju 61453, Republic of Korea; skhkorea@chosun.ac.kr; 5 Department of Emergency Medicine, Chonnam National University Medical School, 42 Jebong-ro, Dong-gu, Gwangju 61469, Republic of Korea

**Keywords:** carbon monoxide poisoning, middle ear barotrauma, hyperbaric oxygen therapy, risk factor

## Abstract

**Background**: Hyperbaric oxygen therapy (HBOT) is the standard treatment for moderate to severe carbon monoxide (CO) poisoning, but middle ear barotrauma (MEB) remains a common complication. This study identified risk factors associated with MEB in patients undergoing monoplace HBOT. **Methods**: This retrospective cohort study included patients treated for CO poisoning with monoplace HBOT at a tertiary academic hospital between May 2021 and December 2023. MEB severity was assessed before and after treatment using video otoscopy and graded according to the modified O’Neill Grading System. **Results**: MEB occurred predominantly at lower severity grades according to the O’Neill scale. In univariate analysis, significant risk factors for MEB included altered mental status at presentation (OR: 3.16, 95% CI: 1.35–7.40, *p* = 0.008), serum albumin > 4.3 g/dL (OR: 0.22, 95% CI: 0.10–0.65, *p* = 0.004), and magnesium levels (OR: 0.21, 95% CI: 0.05–0.98, *p* = 0.046). Multivariate analysis confirmed altered mental status (OR: 3.16, 95% CI: 1.05–9.52, *p* = 0.041), albumin > 4.3 g/dL (OR: 0.26, 95% CI: 0.10–0.65, *p* = 0.004), and magnesium level (OR: 0.21, 95% CI: 0.05–0.88, *p* = 0.033) as independent predictors of MEB. Patients with higher albumin and magnesium levels showed lower risk. **Conclusions**: Altered mental status, lower albumin, and lower magnesium levels predicted middle ear barotrauma in patients undergoing monoplace HBOT for CO poisoning. These findings highlight the importance of careful pre-treatment evaluation and close monitoring during therapy to reduce the incidence of MEB.

## 1. Introduction

Carbon monoxide (CO) poisoning remains a significant public health concern and a leading cause of accidental poisoning deaths worldwide. Globally, the incidence of CO poisoning is estimated at 137 cases per million people, with a reported mortality rate of 4.6 deaths per million people [1]. In South Korea, between 2010 and 2019, a total of 44,361 cases of CO poisoning were identified, corresponding to a prevalence rate of 8.64 cases per 10,000 people. The sources of CO poisoning are varied and encompass residential fires, malfunctioning heating systems, indoor use of charcoal for heating or cooking, and occupational exposure. In South Korea, intentional CO poisoning through charcoal burning has also been identified as a significant cause [2,3]. The high affinity of CO for hemoglobin and the subsequent disruption of oxygen delivery result in severe hypoxia and tissue damage, necessitating prompt and effective treatment [4]. Hyperbaric oxygen therapy (HBOT) is widely regarded as the standard of care for moderate to severe CO poisoning, as it facilitates the rapid elimination of CO from the bloodstream and enhances tissue oxygenation [5].

Despite the well-documented benefits of HBOT in CO poisoning, this treatment is not without risks. Barotrauma, an injury resulting from pressure changes during HBOT, is a notable complication that can affect the ears, sinuses, lungs, or other gas-filled spaces in the body [6,7]. Among these, middle ear barotrauma (MEB) is recognized as the most common complication associated with HBOT, with previous studies reporting incidence ranging from 13.6% to 43.2% [7,8,9]. This condition arises from the inability to equalize pressure between the ambient atmosphere and the middle ear during the pressurization or depressurization phases of treatment. While many cases are mild and self-limiting, severe MEB may result in complications such as persistent ear pain, conductive hearing loss, and, in rare instances, tympanic membrane rupture [9,10].

Given the prevalence of MEB in hyperbaric oxygen therapy, preventive strategies play a crucial role in minimizing its occurrence and ensuring patient safety. One of the most effective methods is patient education regarding pressure-equalizing maneuvers, such as the Valsalva, Toynbee, and Frenzel maneuvers, which promote adequate middle ear ventilation during pressurization. Pressure equalization during middle ear pressurization can be achieved through various maneuvers that facilitate eustachian tube opening. The Valsalva maneuver involves forceful exhalation with a closed mouth and pinched nose, which increases intrathoracic pressure to open the eustachian tubes. In the Toynbee maneuver, swallowing with the nose occluded activates the muscles that dilate the tube. The Frenzel maneuver combines nasal occlusion with coordinated tongue movements to inject air into the middle ear, thereby balancing the pressure. Ensuring that patients can adequately perform these techniques prior to initiating therapy may significantly reduce the risk of MEB [8,11]. Additionally, pre-treatment screening for risk factors such as upper respiratory infections, allergic rhinitis, anatomical variations in the Eustachian tube, ear infections, tympanic membrane perforations, and hearing function should be considered before initiating HBOT to help reduce the risk of MEB [8]. Close monitoring during therapy, including gradual pressurization and depressurization protocols, can further mitigate the likelihood of complications [12]. Nevertheless, preventing HBOT-associated barotrauma remains challenging. Given the potential limitations in applying standard preventive measures, identifying risk factors and determining effective prevention strategies for high-risk patients are crucial. Therefore, this study aims to investigate the risk factors associated with monoplace HBOT-induced barotrauma in patients with CO poisoning.

## 2. Materials and Methods

### 2.1. Study Design

This retrospective cohort study was conducted from May 2021 to December 2023 at Chonnam National University using our monoplace HBOT chamber (BARA-MED Monoplace Hyperbaric Chamber, ETC Biomedical Systems, Southampton, PA, USA). We used a monoplace HBOT chamber, which is designed to accommodate a single patient in a controlled hyperbaric environment. This chamber administers 100% medical-grade oxygen at increased atmospheric pressure, thereby enhancing tissue oxygenation. Its transparent acrylic construction allows for continuous visual monitoring, ensuring patient safety and precise management of treatment conditions. Each HBOT session lasted approximately 90 min, following a modified protocol based on the standard CO intoxication treatment [13]. Unlike the standard approach, our protocol was adapted for a monoplace hyperbaric chamber system, which does not reach 3.0 atmospheres absolute (ATA). The session began with a 15 min compression phase to increase the pressure to 2.8 ATA. This was followed by a 30 min treatment period at 2.8 ATA, after which the pressure was gradually decreased to 2.0 ATA over 5 min. The session continued with a 30 min treatment period at 2.0 ATA, followed by decompression to ambient pressure over a 10 min interval to conclude the therapy.

Medical records were retrospectively collected and analyzed. The primary objective was to assess the incidence of MEB among the treated patients. Secondary objectives included identifying patient-related risk factors, such as demographic characteristics, physiological parameters, detailed medical history (including history of suicide attempts), and blood test results, which might be associated with the occurrence of MEB. A video otoscope (INSIGHT-I^®^, MEDIANA, Wonju, Republic of Korea) was utilized to obtain baseline images of the tympanic membrane (TM) before hyperbaric exposure to assess for MEB. These images served as reference points for subsequent evaluations of MEB severity according to the modified O’Neill Grading System [14]. The severity of MEB can be categorized based on otologic findings. Grade 0 represents the absence of any otologic signs of trauma. Grade 1 is characterized by increased redness of the TM compared with baseline, the presence of serous or mildly serosanguineous fluid, and/or evidence of trapped air behind the TM. Grade 2 involves frank bleeding in any location and/or perforation of the TM. For the purposes of this study, Grades 1 and 2 were classified as MEB, indicating objective signs of barotrauma affecting the middle ear. This study was approved by the Chonnam National University Hospital Institutional Review Board (CNUH-2023-305). Due to its retrospective nature, informed consent was waived.

### 2.2. Participants

The study included all eligible patients who presented to the emergency department (ED) with CO poisoning, diagnosed based on patient history, and received HBOT during the study period. According to institutional criteria, patients were considered candidates for HBOT if they exhibited any of the following: a history of loss of consciousness, neurocognitive symptoms (e.g., headache, dizziness), elevated cardiac enzyme levels, or the initial carboxyhemoglobin (COHb) level ≥ 25% [5].

### 2.3. Data Collection

Data for this study were retrospectively collected from medical records of patients who presented to the ED with carbon monoxide poisoning and subsequently underwent HBOT at our institution. Demographic data, including age and sex, were recorded, along with detailed medical and medication histories, incorporating conditions such as hypertension, diabetes mellitus, cancer, pregnancy, liver cirrhosis, chronic kidney disease, and cerebrovascular and cardiovascular diseases, as well as otitis media and rhinitis. In addition, the documented clinical parameters included the level of consciousness, elapsed time from CO exposure to initiation of HBOT, and laboratory data. The laboratory parameters analyzed were as follows: white blood cell (WBC) count, hemoglobin, platelet count, blood urea nitrogen (BUN), creatinine, aspartate aminotransferase (AST), alanine aminotransferase (ALT), albumin, C-reactive protein (CRP), glucose, creatine kinase (CK), sodium, potassium, chloride, calcium, magnesium, initial COHb level, and troponin I. Complications related to HBOT—including MEB, nasal sinus pain, ocular complications, and claustrophobia—were noted. Claustrophobia, in this context, refers to a fear or anxiety reaction triggered by confinement within the enclosed HBOT chamber. Post-HBOT otoscopic examinations were performed using the O’Neill scale for assessment and grading.

### 2.4. Statistical Analysis

Continuous variables were initially assessed for normality using skewness and kurtosis. Variables meeting the normality assumption are presented as means ± standard deviations, whereas non-normally distributed variables are reported as medians with interquartile ranges (IQRs). Categorical variables are summarized as frequencies with corresponding percentages. Comparisons between independent groups were conducted using either the *t*-test or Mann–Whitney U test for continuous variables, depending on their distribution, and the Chi-squared test or Fisher’s exact test for categorical variables. To evaluate relationships between continuous variables and the outcome, restricted cubic spline (RCS) curves were generated to visualize the probability of MEB occurrence across different values. Based on these spline curves, variables exhibiting a nonlinear relationship with MEB occurrence were categorized into two groups according to identified cutoff values.

**Figure A1:**
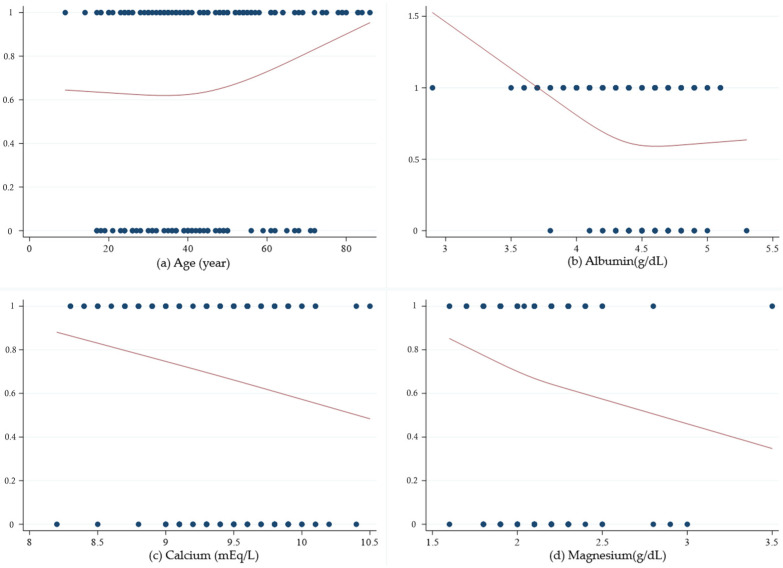
Restricted cubic spline plots showing the associations between serum age, albumin, calcium (Ca), and magnesium (Mg) levels and the occurrence of middle ear barotrauma in patients with carbon monoxide poisoning undergoing hyperbaric oxygen therapy. Each panel demonstrates the fitted relationship (solid red line) and the observed data (blue dots), with three knots used for spline fitting. (**a**) Age: a U-shaped trend with increasing risk in older age groups (R^2^ (Coefficient of Determination) = 0.0318; RMSE (Root Mean Squared Error) = 0.4645). (**b**) Serum albumin: a nonlinear negative association was most prominent among lower albumin levels (R^2^ = 0.0856; RMSE = 0.4506). (**c**) Serum calcium: a slight negative association was observed (R^2^ = 0.0294; RMSE = 0.465). (**d**) Serum magnesium: a mild inverse relationship was noted (R^2^ = 0.0229; RMSE = 0.4666). Methodological Note: Dots represent individual observations. Solid red lines indicate the fitted nonlinear trend estimated using logistic regression. Knots for each spline were placed at three quantiles.

Specifically, age and albumin demonstrated a nonlinear association with the outcome, prompting their categorization into dichotomous groups: age (≤50 vs. >50 years) and albumin (≤4.3 vs. >4.3 g/dL). Conversely, calcium (Ca) and magnesium (Mg) displayed a consistent linear association with decreased MEB probability; thus, these variables were retained as continuous predictors in the model (Appendix A Figure A1). Univariate logistic regression analysis was performed to select variables; those with a *p*-value < 0.1 were subsequently included in the multivariate logistic regression model to identify independent predictors of MEB occurrence in HBOT. Logistic regression results are reported as odds ratios (ORs) with corresponding 95% confidence intervals (CIs). Statistical analyses were conducted using Stata/SE version 16.1 (StataCorp, College Station, TX, USA), with two-sided *p*-values < 0.05 considered statistically significant.

## 3. Results

**Figure 1:**
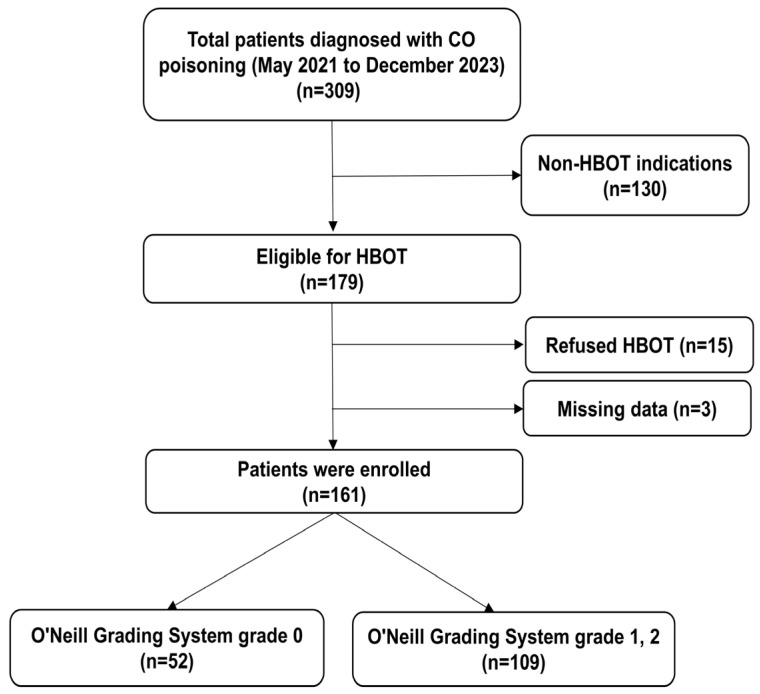
Middle ear barotrauma in patients with CO poisoning undergoing monoplace HBOT. HBOT—hyperbaric oxygen therapy; CO—carbon monoxide.

Among 309 patients diagnosed with CO poisoning, 130 did not meet the institutional indications for HBOT and were therefore excluded. An additional 15 patients who met the indications declined treatment, and 3 patients were excluded due to incomplete records. After these exclusions, a total of 161 patients were included in the final analysis (Figure 1).

The median age of the included patients was 43 years (IQR, 32–55). The majority were male (74.5%), and the median time from symptom onset to treatment was 3.50 h (IQR, 2.33–5.92), with 41.0% receiving treatment within 3 h. Upon arrival at the ED, 69.8% of patients were fully alert, while 30.2% exhibited altered mental status. The median systolic and diastolic blood pressures were 120 mmHg and 70 mmHg, respectively. The median body temperature was 36.5 °C and the median oxygen saturation was 98%. Notably, 34.6% of patients experienced CO intoxication due to intentional self-harm or suicide attempts. The most common comorbidities were hypertension (12.4%), diabetes (6.8%), and cancer (3.7%). Smoking history was present in 40.4% of patients and 19.9% had psychiatric disorders. Laboratory findings revealed a median white blood cell (WBC) count of 9800/μL, hemoglobin level of 15 g/dL, and albumin level of 4.4 g/dL. Median sodium, potassium, Ca, and Mg levels were 140 mEq/L, 3.9 mEq/L, 9.5 mg/dL, and 2.1 mg/dL, respectively. The initial COHb level was 11.9% (Table 1).

Among patient-reported HBOT complications, otalgia was the most frequent (40.4%), followed by nasal sinus pain (0.6%) and claustrophobia (1.2%). Meanwhile, 57.8% of patients reported no subjective complications. Based on the O’Neill Grading System, 32.3% were classified as Grade 0, 65.8% as Grade 1, and 1.9% as Grade 2 (Table 2).

Patients who developed MEB (O’Neill grades 1–2) had a higher mean age (46.57 ± 18.24 years) compared to those without MEB (40.83 ± 14.45 years, *p* = 0.048). Although there was no statistically significant difference in sex distribution, the proportion of females was higher in the MEB group (28.4% vs. 19.2%). Additionally, 34.6% of patients in this study experienced CO intoxication due to self-harm intent, with no significant difference between groups. Among clinical factors, the incidence of altered mental status upon ED arrival was significantly greater in the MEB group (37.0% vs. 15.7%, *p* = 0.006). Patients who received treatment within 3 h had a higher proportion of MEB cases (45.9% vs. 30.8%, *p* = 0.068). Regarding laboratory findings, patients with MEB had significantly lower albumin levels than those without MEB (4.33 ± 0.40 g/dL vs. 4.53 ± 0.25 g/dL, *p* < 0.001). Additionally, Ca (9.35 ± 0.47 mg/dL vs. 9.52 ± 0.43 mg/dL, *p* = 0.031) and Mg (2.08 ± 0.25 mg/dL vs. 2.16 ± 0.26 mg/dL, *p* = 0.039) levels were lower in the MEB group, whereas Troponin I was higher (0.33 ± 1.14 ng/mL vs. 0.18 ± 0.64 ng/mL, *p* = 0.023). Other laboratory markers, including the initial COHb level, inflammatory indicators, and renal function tests, showed no statistically significant differences. Among complications related to HBOT, otalgia was more frequent in the MEB group (57.8% vs. 3.8%). Claustrophobia rates were similar in both groups (Table 3).

In the univariate logistic regression analysis, several variables emerged as statistically significant predictors of MEB. Patients older than 50 years had increased odds of developing MEB (OR: 3.00, 95% CI: 1.33–6.77, *p* = 0.008). Furthermore, patients presenting with altered mental status to the ED (VPU: verbal/painful/unresponsive) had elevated odds of MEB compared to alert patients (OR: 3.16, 95% CI: 1.35–7.40, *p* = 0.008). Lower albumin levels (<4.3 g/dL) were similarly associated with a higher risk of MEB (OR: 0.22, 95% CI: 0.10–0.48, *p* < 0.001), as were lower Ca (OR: 0.44, 95% CI: 0.20–0.94, *p* = 0.034) and lower Mg levels (OR: 0.29, 95% CI: 0.08–1.11, *p* = 0.070), although the latter relationship was only borderline significant. Some of these associations retained statistical significance in the multivariate logistic regression analysis. Patients with altered mental status remained significantly associated with MEB (OR: 2.72, 95% CI: 1.04–7.12, *p* = 0.041). Additionally, lower albumin levels continued to significantly predict MEB (OR: 0.26, 95% CI: 0.10–0.65, *p* = 0.004), as did lower Mg levels (OR: 0.21, 95% CI: 0.05–0.98, *p* = 0.046). However, the effect of age greater than 50 years was no longer statistically significant (OR: 2.34, 95% CI: 0.91–6.02, *p* = 0.078). Likewise, lower Ca levels were not significant in the multivariate model (OR: 1.22, 95% CI: 0.46–3.21, *p* = 0.694) (Table 4).

## 4. Discussion

In our study, MEB occurred in 67.7% of patients, which is higher than the rates reported in previous studies, ranging from 13.6% to 43.2%. This discrepancy may be attributed to differences in chamber type (monoplace vs. multiplace), compression rates, and diagnostic criteria. Notably, studies using monoplace chambers with rapid pressurization protocols and objective assessments such as video otoscopy tend to report higher MEB incidence compared to those relying solely on self-reported symptoms [7,8,9]. A prior study suggested that monoplace HBOT may increase the risk of MEB, particularly during the compression phase, due to elevated central venous pressure and venous congestion in the supine position, along with rapid oxygen absorption into the middle ear under 100% oxygen pressurization [8]. Despite these factors, the incidence of MEB among CO-intoxicated patients appears higher than among other patient populations, suggesting that unique pathophysiological characteristics of CO poisoning may further predispose someone to increased susceptibility to MEB. Patients presenting with impaired consciousness demonstrated a significantly higher risk of developing MEB following HBOT. Although we did not assess the actual implementation or effectiveness of pressure-equalizing maneuvers, it is plausible that patients with altered mental status may have difficulty understanding instructions or performing such maneuvers, thereby increasing their vulnerability to MEB. Techniques such as the Valsalva, Toynbee, and Frenzel maneuvers are known to promote middle ear ventilation and prevent barotrauma; thus, an inability to perform them could be one of several contributing mechanisms. Furthermore, decreased responsiveness in such patients may lead to delayed symptom recognition and reduced chances for early intervention. These interpretations are hypothetical and warrant further study but underscore the need for enhanced monitoring and individualized preventive strategies in patients with impaired consciousness undergoing HBOT.

Our study identified a significant protective effect of albumin against MEB in patients with CO intoxication undergoing HBOT. Patients with albumin levels above 4.3 g/dL exhibited a 74% reduction in MEB risk (OR = 0.26, 95% CI: 0.10–0.65), suggesting a potential role of albumin in mitigating pressure-induced ear injuries. This finding is particularly notable, as no prior studies have directly linked albumin levels with the prevention of HBOT-induced barotrauma in patients with CO intoxication. One possible explanation for this protective effect is albumin’s role in maintaining vascular integrity and microcirculatory stability. Albumin is a key regulator of colloid osmotic pressure, preventing excessive fluid leakage from capillaries into surrounding tissues [15,16]. During HBOT, rapid pressure fluctuations can induce vascular stress and microvascular leakage in the middle ear mucosa, potentially leading to edema and impaired pressure equalization [17]. Higher albumin levels may enhance vascular resilience, thereby minimizing fluid shifts and preventing barotrauma. Previous studies have demonstrated that low albumin levels are associated with increased vascular permeability and tissue edema in critically ill patients, supporting this hypothesis [18]. Another possible mechanism involves albumin’s anti-inflammatory and antioxidant properties [19,20]. CO intoxication induces oxidative stress and systemic inflammation, leading to endothelial dysfunction and increased vascular permeability [4]. Several studies have highlighted albumin’s antioxidant and anti-inflammatory properties, which play a crucial role in mitigating oxidative stress and vascular dysfunction. Albumin neutralizes free radicals, protects antioxidants, and acts as a transporter for antioxidant molecules [19,21,22]. Additionally, albumin interacts with inflammatory mediators, modulating immune responses and reducing inflammation [23,24]. By reducing oxidative stress and modulating inflammatory pathways, albumin may help maintain the structural integrity of the middle ear mucosa, thereby lowering the risk of HBOT-induced barotrauma.

Along with albumin, Mg was also associated with a reduced risk of MEB in our study. Although both Ca and Mg were significant in the univariate analysis, only Mg remained statistically significant in the multivariate logistic regression model, suggesting that its effect is independent of albumin levels and other confounding factors. To date, no studies have directly investigated the relationship between Mg levels and the risk of MEB. However, given Mg’s physiological functions, its role in vascular regulation, inflammation control, and oxidative stress modulation may plausibly contribute to the observed protective effect. Mg is a calcium antagonist, promoting vasodilation and preventing excessive vasoconstriction, thus helping to maintain microcirculatory stability in the mucosa [25,26]. This effect may reduce edema and optimize Eustachian tube function, facilitating pressure equalization during HBOT. Additionally, Mg has been shown to modulate inflammatory pathways by suppressing cytokines, such as IL-6 and TNF-α, while also acting as an antioxidant [27], mitigating oxidative damage that could compromise middle ear integrity [28]. While these mechanisms offer a potential explanation for our findings, further research is required to determine whether Mg supplementation or targeted Mg level optimization could serve as an effective preventive strategy against HBOT-induced barotrauma.

Although initial COHb level was recorded and included in the analysis, it was not a statistically significant predictor of MEB. This suggests that, rather than the absolute COHb level, individual physiological responses, such as the degree of neurological impairment or metabolic factors, may play a more critical role in the development of barotrauma. The relatively high incidence of MEB among CO-intoxicated patients may therefore be associated with such patient-specific factors, which warrants further investigation.

This study has several limitations. Firstly, this was a retrospective, single-center study, which inherently limits the ability to establish causal relationships and to generalize the findings to broader populations. Multicenter prospective studies are required to validate these results and enhance external validity. Secondly, this study was not a randomized controlled trial design, making it susceptible to potential confounding factors that were not fully accounted for in the analysis. Thirdly, long-term follow-up data regarding MEB-related complications were not collected, thus preventing the evaluation of potential chronic effects of barotrauma in CO-intoxicated patients undergoing HBOT. Fourthly, all patients were treated in a monoplace hyperbaric chamber, which differs in several aspects—including patient positioning, ventilation, and patient interaction with medical staff—from multiplace chamber environments. These differences may limit the generalizability of our findings to other HBOT settings. Additionally, the treatment protocol used in this study was adapted to the technical limitations of the monoplace chamber. While the protocol described by Weaver et al. involves 3.0 ATA, it was conducted at high altitude, and in most sea-level regions, 2.8 ATA is commonly used as an equivalent standard. Given the absence of a universally accepted protocol for monoplace HBOT in CO poisoning, such variability limits direct comparisons across studies, and its potential impact on MEB incidence remains uncertain. Fifthly, individual anatomical variability in Eustachian tube function and potential pre-existing otologic conditions were not assessed; such factors may have influenced patient susceptibility to MEB. Lastly, the relatively small sample size in our study may limit the precision of the estimated MEB incidence and increase the potential for sampling variability. Larger, multicenter studies are warranted to confirm the generalizability and robustness of our findings.

## 5. Conclusions

In conclusion, this study highlights the high incidence of MEB in CO-intoxicated patients undergoing monoplace HBOT, particularly among individuals with altered mental status. Our findings suggest that careful pre-treatment evaluation and close monitoring during therapy are essential for reducing the risk of MEB. Additionally, metabolic factors such as albumin and magnesium levels may influence susceptibility and, therefore, merit further investigation. Future research should focus on refining preventive strategies and assessing their applicability across diverse clinical settings.

## Figures and Tables

**Table 1:** Baseline characteristics of patients with CO intoxication treated with monoplace hyperbaric oxygen therapy.

Variable	*n* (%) or Median (IQR)	Min	Max
Total patients (*n*)	161	-	-
Age (years)	43 (32–55)	9	86
Sex			
Male	120 (74.5)	-	-
Female	41 (25.5)	-	-
Time from onset to treatment (hours)	3.50 (2.33–5.92)	1	150.17
Time from onset to treatment (categorized)			
≤3 h	66 (41.0)	-	-
>3 h	95 (59.0)	-	-
Mental status at ED arrival (*n* = 159)			
Alert (A)	111 (69.8)	-	-
Verbal response (V)	27 (17.0)	-	-
Pain response (P)	13 (8.2)	-	-
Unresponsive (U)	8 (5.0)	-	-
Mental status at ED arrival (grouped) (*n* = 159)			
Alert (A)	111 (69.8)	-	-
Impaired (VPU)	48 (30.2)	-	-
Systolic blood pressure (mmHg) (*n* = 158)	120 (110–140)	70	180
Diastolic blood pressure (mmHg) (*n* = 158)	70 (60–80)	40	100
Body temperature (°C) (*n* = 158)	36.5 (36.2–36.9)	34	38.5
Heart rate (bpm) (*n* = 158)	92 (80–108)	12	168
Oxygen saturation (%) (*n* = 152)	98 (97–99)	88	100
Suicide attempt (*n* = 159)	55 (34.6)	-	-
Past medical history			
Cardiac disease	11 (6.8)	-	-
Hypertension	20 (12.4)	-	-
Diabetes mellitus	11 (6.8)	-	-
Cerebrovascular disease	2 (1.2)	-	-
Chronic respiratory disease	3 (1.9)	-	-
Cancer	6 (3.7)	-	-
Chronic liver disease	3 (1.9)	-	-
Chronic kidney disease	2 (1.2)	-	-
Otorhinolaryngologic condition			
Rhinitis	1 (0.6)	-	-
Psychiatric disorder	32 (19.9)	-	-
Smoking history	65 (40.4)	-	-
Other medical condition	32 (19.9)	-	-
Laboratory findings			
WBC (×10^3^/µL) (*n* = 159)	9800 (7500–13500)	11.6	31,900
Hemoglobin (g/dL) (*n* = 159)	15 (13.4–16)	7.9	18.6
Platelet count (×10^3^/µL) (*n* = 159)	256 (200–313)	55	506
BUN (mg/dL) (*n* = 159)	13.1 (10.8–17.6)	5.6	59.4
Creatinine (mg/dL) (*n* = 159)	0.81 (0.70–0.99)	0.43	2.69
AST (U/L) (*n* = 159)	26 (21–36)	4	553
ALT (U/L) (*n* = 159)	22 (14–34)	5	152
Albumin (g/dL) (*n* = 159)	4.4 (4.1–4.7)	2.9	5.3
CRP (mg/dL) (*n* = 159)	0.08 (0.03–0.30)	0.001	22
Glucose (mg/dL) (*n* = 159)	111 (96–139)	63	963
CK (U/L) (*n* = 159)	129 (84–265)	42	29,459
Sodium (mEq/L) (*n* = 159)	140 (139–142)	14.2	146
Potassium (mEq/L) (*n* = 159)	3.9 (3.7–4.2)	0.34	5.8
Chloride (mEq/L) (*n* = 159)	105 (103–106)	10.9	114
Calcium (mg/dL) (*n* = 158)	9.5 (9.1–9.8)	8.2	10.5
Magnesium (mg/dL) (*n* = 158)	2.1 (1.9–2.2)	1.6	3.5
Initial COHb level (%) (*n* = 160)	11.9 (4.25–22.55)	2.3	50.9
Troponin I (*n* = 157)	0.009 (0.003–0.091)	0.002	8.745

IQR—interquartile range; CO—carbon monoxide; ED—emergency department; WBC—white blood cell count; BUN—blood urea nitrogen; AST—aspartate aminotransferase; ALT—alanine aminotransferase; CRP—C-reactive protein; CK—creatine kinase; COHb—carboxyhemoglobin.

**Table 2:** Monoplace hyperbaric oxygen therapy-related complications based on subjective symptoms and O’Neill grading.

Variable	*n* (%)
HBOT complications	
Otalgia	65 (40.4)
Nasal sinus pain	1 (0.6)
Claustrophobia	2 (1.2)
No complication	93 (57.8)
O’Neill Grading System grade	
0	52 (32.3)
1	106 (65.8)
2	3 (1.9)

HBOT—hyperbaric oxygen therapy. O’Neill Grading System: Grade 0 = no trauma; Grade 1 = tympanic membrane redness, fluid, or trapped air; Grade 2 = bleeding or tympanic membrane perforation.

**Table 3:** Comparison of clinical and laboratory factors associated with middle ear barotrauma in CO-intoxicated patients undergoing monoplace hyperbaric oxygen therapy.

	Middle Ear Barotrauma (O’Neill Grading System)		
Variable	Grade 0	Grade 1–2	*p*-Value
Number of patients, *n*	52	109	
Age (years)	40.83 ± 14.45	46.57 ± 18.24	0.048
Sex			0.210
Male	42 (80.8)	78 (71.6)	
Female	10 (19.2)	31 (28.4)	
Time from onset to treatment (hours)	7.33 ± 13.72	6.28 ± 14.55	0.194
Time from onset to treatment (categorized)			0.068
≤3 h	16 (30.8)	50 (45.9)	
>3 h	36 (69.2)	59 (54.1)	
Mental status at admission (*n* = 159)			0.062
Alert (A)	43 (84.3)	68 (63.0)	
Verbal response (V)	5 (9.8)	22 (20.4)	
Pain response (P)	2 (3.9)	11 (10.2)	
Unresponsive (U)	1 (2.0)	7 (6.5)	
Mental status at ED arrival (grouped) (*n* = 159)			0.006
Alert (A)	43 (84.3)	68 (63.0)	
Impaired (VPU)	8 (15.7)	40 (37.0)	
Systolic blood pressure (mmHg) (*n* = 158)	124.86 ± 16.75	121.13 ± 22.42	0.245
Diastolic blood pressure (mmHg) (*n* = 158)	73.78 ± 10.09	71.91 ± 12.70	0.356
Body temperature (°C) (*n* = 158)	36.53 ± 0.47	36.58 ± 0.56	0.874
Heart rate (bpm) (*n* = 158)	93.35 ± 19.27	94.57 ± 20.63	0.407
Oxygen saturation (%) (*n* = 152)	98.17 ± 1.15	97.68 ± 2.16	0.493
Suicide attempt (*n* = 159)	33 (63.5)/19 (36.5)	71 (66.4)/36(33.6)	0.719
Past medical history			
Cardiac disease	50 (96.2)/2 (3.8)	100 (91.7)/9 (8.3)	0.505
Hypertension	46 (88.5)/6 (11.5)	95 (87.2)/14 (12.8)	0.814
Diabetes mellitus	48 (92.3)/4 (7.7)	102 (93.6)/7 (6.4)	0.748
Cerebrovascular disease	52 (100.0)/0 (0.0)	107 (98.2)/2 (1.8)	1.000
Chronic respiratory disease	51 (98.1)/1 (1.9)	107 (98.2)/2 (1.8)	1.000
Cancer	52 (100.0)/0 (0.0)	103 (94.5)/6 (5.5)	0.178
Chronic liver disease	52 (100.0)/0 (0.0)	106 (97.3)/3 (2.7)	0.552
Chronic kidney disease	51 (98.1)/1 (1.9)	108 (99.1)/1 (0.9)	0.543
Psychiatric disorders	42 (80.8)/10 (19.2)	87 (79.8)/22 (20.2)	0.887
Smoking history	29 (55.8)/23 (44.2)	67 (61.5)/42 (38.5)	0.491
Other medical conditions	45 (86.5)/7 (13.5)	84 (77.1)/25(22.9)	0.159
Laboratory findings			
WBC (×10^3^/µL) (*n* = 159)	10,594.35 ± 4497.83	10,808.61 ± 5325.40	0.859
Hemoglobin (g/dL) (*n* = 159)	14.94 ± 2.02	14.67 ± 1.82	0.182
Platelet count (×10^3^/µL) (*n* = 159)	275.90 ± 70.32	256.64 ± 83.06	0.083
BUN (mg/dL) (*n* = 159)	13.66 ± 4.96	15.05 ± 7.07	0.269
Creatinine (mg/dL) (*n* = 159)	0.87 ± 0.23	0.86 ± 0.30	0.359
AST (U/L) (*n* = 159)	39.16 ± 50.13	43.12 ± 62.02	0.133
ALT (U/L) (*n* = 159)	27.90 ± 21.58	29.89 ± 25.26	0.765
Albumin (g/dL) (*n* = 159)	4.53 ± 0.25	4.33 ± 0.40	<0.001
CRP (mg/dL) (*n* = 159)	0.99 ± 3.64	0.56 ± 1.42	0.937
Glucose (mg/dL) (*n* = 159)	123.31 ± 50.64	136.96 ± 97.16	0.275
Calcium (mg/dL) (*n* = 158)	9.52 ± 0.43	9.35 ± 0.47	0.031
CK (U/L) (*n* = 159)	1038.08 ± 4445.12	594.21 ± 2214.50	0.868
Sodium (mEq/L) (*n* = 159)	138.00 ± 17.85	140.08 ± 2.78	0.506
Potassium (mEq/L) (*n* = 159)	3.99 ± 0.35	3.93 ± 0.58	0.434
Chloride (mEq/L) (*n* = 159)	104.59 ± 2.64	103.55 ± 9.57	0.596
Magnesium (mg/dL) (n = 158)	2.16 ± 0.26	2.08 ± 0.25	0.039
Initial COHb level (%) (n = 160)	13.24 ± 9.40	14.39 ± 11.69	0.910
Troponin I (ng/mL) (*n* = 157)	0.18 ± 0.64	0.33 ± 1.14	0.023
HBOT complication			<0.001
Otalgia	2 (3.8)	63 (57.8)	
Nasal sinus pain	0 (0.0)	1 (0.9)	
Claustrophobia	1 (1.9)	1 (0.9)	
No complication	49 (94.2)	44 (40.4)	

HBOT—hyperbaric oxygen therapy; CO—carbon monoxide; ED—emergency department; WBC—white blood cell count; BUN—blood urea nitrogen; AST—aspartate aminotransferase; ALT—alanine aminotransferase; CRP—C-reactive protein; CK—creatine kinase; COHb—carboxyhemoglobin.

**Table 4:** Logistic regression analysis of factors associated with middle ear barotrauma in CO-intoxicated patients undergoing monoplace hyperbaric oxygen therapy.

Variable	Univariate OR (95% CI)	*p*-Value	Multivariate OR (95% CI)	*p*-Value
Age	1.02 (1.00–1.04)	0.051	-	-
Age Group				
≤50 years	1 (Reference)	-	1 (Reference)	-
>50 years	3.00 (1.33–6.77)	0.008	2.34 (0.91–6.02)	0.078
Sex				
Male	1 (Reference)	-	-	-
Female	1.67 (0.75–3.74)	0.212	-	-
Time from onset to treatment (hours)	0.99 (0.97–1.02)	0.665	-	-
Time from onset to treatment (grouped)				
≤3 h	1 (Reference)	-	1 (Reference)	-
>3 h	0.52 (0.26–1.06)	0.070	0.59 (0.27–1.31)	0.198
Mental status at ED arrival (*n* = 159)				
Alert (A)	1 (Reference)	-	1 (Reference)	-
Impaired(VPU, verbal/painful/unresponsive)	3.16 (1.35–7.40)	0.008	2.72 (1.04–7.12)	0.041
Systolic blood pressure (*n* = 158)	0.99 (0.98–1.01)	0.291	-	-
Diastolic blood pressure (*n* = 158)	0.99 (0.96–1.02)	0.354	-	-
Suicide attempt (*n* = 159)	0.88 (0.44–1.76)	0.719	-	-
Past medical history				
Cardiac disease	2.25 (0.47–10.81)	0.311	-	-
Hypertension	1.13 (0.41–3.13)	0.814	-	-
Diabetes mellitus	0.82 (0.23–2.95)	0.765	-	-
Cerebrovascular disease	-	-	-	-
Chronic respiratory disease	0.95 (0.08–10.76)	0.969	-	-
Cancer	-	-	-	-
Chronic liver disease	-	-	-	-
Chronic kidney disease	0.47 (0.03–7.70)	0.598	-	-
Psychiatric history	1.06 (0.46–2.44)	0.887	-	-
Smoking history	0.79 (0.40–1.54)	0.491	-	-
Other condition	1.91 (0.77–4.77)	0.164	-	-
Laboratory findings				
WBC (*n* = 159)	1.00 (1.00–1.00)	0.803	-	-
Hemoglobin (g/dL) (*n* = 159)	0.92 (0.77–1.11)	0.398	-	-
Platelet count (×10^3^/µL) (*n* = 159)	1.00 (0.99–1.00)	0.156	-	-
BUN (*n* = 159)	1.04 (0.98–1.11)	0.214	-	-
Creatinine (*n* = 159)	0.87 (0.27–2.83)	0.815	-	-
AST (*n* = 159)	1.00 (0.99–1.01)	0.691	-	-
ALT (*n* = 159)	1.00 (0.99–1.02)	0.627	-	-
Albumin (*n* = 159)	0.18 (0.06–0.54)	0.002		
≤4.3 g/dL	1 (Reference)	-	1 (Reference)	-
>4.3 g/dL	0.22 (0.10–0.48)	<0.001	0.26 (0.10–0.65)	0.004
Calcium (*n* = 158)	0.44 (0.20–0.94)	0.034	1.22 (0.46–3.21)	0.694
Magnesium (*n* = 158) Initial COHb level (%) (*n* = 160)	0.29 (0.08–1.11) 1.01 (0.98–1.04)	0.070 0.533	0.21 (0.05–0.98)	0.046
Troponin I (ng/mL) (*n* = 157)	1.22 (0.78–1.91)	0.390		

OR—odds ratio; CI—confidence interval; CO—carbon monoxide; ED—emergency department; WBC—white blood cell count; BUN—blood urea nitrogen; AST—aspartate aminotransferase; ALT—alanine aminotransferase; COHb—carboxyhemoglobin.

## Data Availability

The datasets collected and analyzed in the current study are available from the corresponding author on reasonable request.

## Abbreviations

The following abbreviations are used in this manuscript:

HBOT	Hyperbaric Oxygen Therapy
CO	Carbon Monoxide
MEB	Middle Ear Barotrauma
COHb	Carboxyhemoglobin
ED	Emergency Department
TM	Tympanic Membrane
ATA	Atmospheres Absolute
WBC	White Blood Cell Count
BUN	Blood Urea Nitrogen
AST	Aspartate Aminotransferase
ALT	Alanine Aminotransferase
CRP	C-Reactive Protein
CK	Creatine Kinase
OR	Odds Ratio
CI	Confidence Interval
IQR	Interquartile Range
